# Supplementary material for: A yeast one-hybrid and microfluidics-based pipeline to map mammalian gene regulatory networks
Source: Mol Syst Biol. 2013 Aug 6;9:682. doi: 10.1038/msb.2013.38 (PMC3779800; doi:10.1038/msb.2013.38)
Supplement: Supplementary Information — Supplementary Figures S1-S19 [file msb201338-s1.pdf]

Supplementary Figures

A yeast one-hybrid and microfluidics-based pipeline to map mammalian gene regulatory networks

Carine Gubelmann[1], Sebastian M. Waszak[1], Alina Isakova[1], Wiebke Holcombe[1], Korneel Hens[1], Antonina Iagovitina[1], Jean-Daniel Feuz[1], Sunil K. Raghav[1], Jovan Simicevic[1], Bart Deplancke[1§]

Supplementary Figures S1-7. Representative TIDY-processed images for each Y1H screen by transformation and mating.
Supplementary Figure S8. Manual transformation and mating of the reproducibly detected interactions in the Y1H screens.
Supplementary Figure S9. *In silico* predicted nucleosome landscape for DNA elements screened with Y1H.
Supplementary Figure S10. Luciferase assay results.
Supplementary Figure S11. Scheme of the pMARE vector.
Supplementary Figure S12. Relative DNA occupancy landscape for RFX2 on the *Mcts2-Id1* enhancer element with two MARE approaches: on-chip protein expression and off-chip synthesis using a multiplexer design.
Supplementary Figure S13. Identification of specific protein-DNA interactions with MARE on the *Mmp9* promoter element with consensus TFs.
Supplementary Figure S14. PWM scores and the relative DNA occupancy landscape of the *Mmp9* promoter.
Supplementary Figure S15. PWM scores and the relative DNA occupancy landscape of the *Mcts2-Id1* enhancer.
Supplementary Figure S16. Identification of specific protein-DNA interactions with MARE on the *Mcts2-Id1* enhancer element with consensus TFs.
Supplementary Figure S17. ChIP-qPCR validations of SP3, SP4, and NFKB1 binding to the *Mmp9* promoter in TNF-α stimulated NIH-3T3 cells.
Supplementary Figure S18. Comparative sequence analysis of the *Mcts2-Id1* enhancer element.
Supplementary Figure S19. LacZ staining results of the transgenic embryos bearing the *Mcts2-Id1* enhancer element with or without the deletion.

A

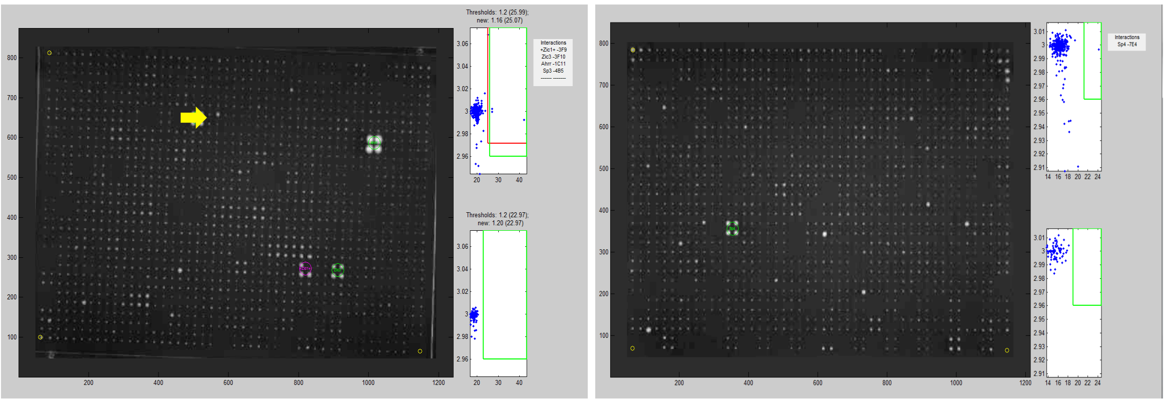

B

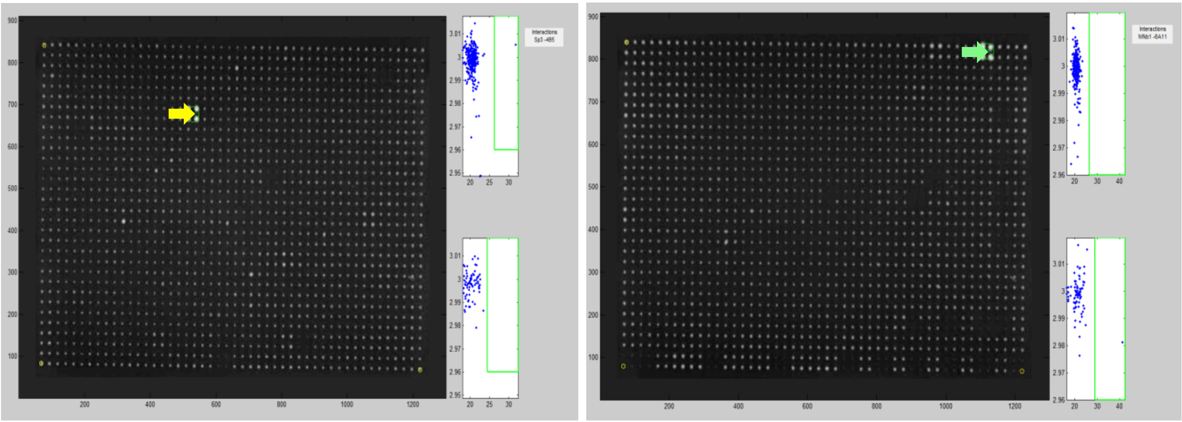

Figure S1 ¦ Y1H-based interactions observed with the *Mmp9* promoter based on growth on a selective plate using either (**A**) the direct transformation approach (haploid yeast strains) with 20mM 3-Amino-1,2,4-triazole (3-AT) or (**B**) the mating approach (diploid yeast) with 5mM 3-AT. The yellow and green arrows point to the previously reported TF interactors SP3 and NFKB1 respectively. The identity of each positive TF can be found in **Supplementary Table S3**. The green circles represent the interactions scoring 20% above the highest background intensity value (default). Those indicated in magenta are interactions that bordered the cluster of called positives and that as such still clearly exhibited superior growth than highest background levels.

A

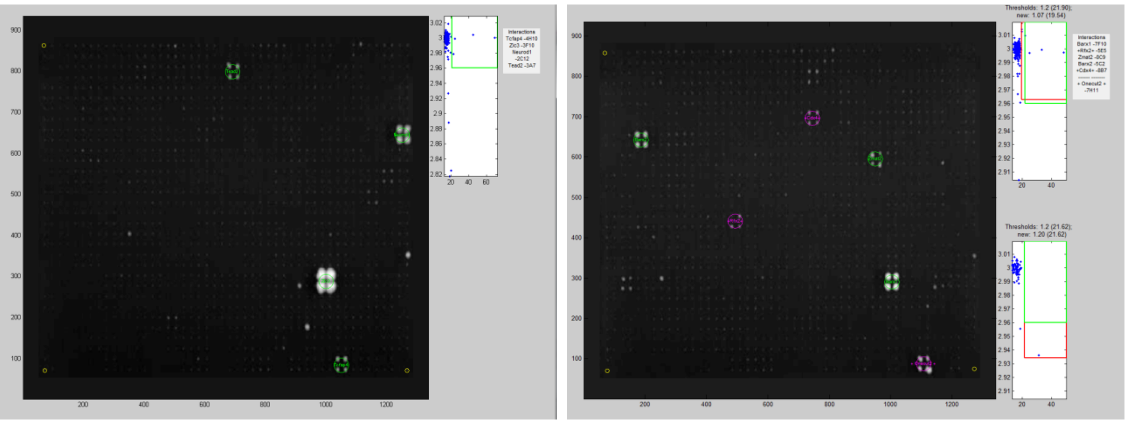

B

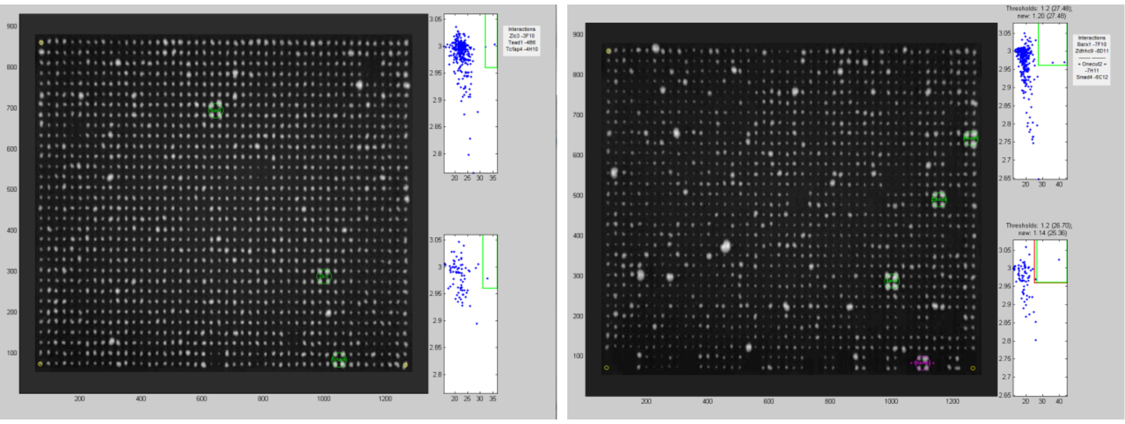

Figure S2 ¦ Y1H-based interactions observed with the *Rhs7-2* DNA bait based on growth on a selective plate using either (**A**) the direct transformation approach (haploid yeast strains) with 20mM 3-Amino-1,2,4-triazole (3-AT) or (**B**) the mating approach (diploid yeast) with 5mM 3-AT. The identity of each positive interaction can be found in **Supplementary Table S3**. One representative experiment is shown for each Y1H procedure. The green circles represent the interactions scoring 20% above the highest background intensity value (default). Those indicated in magenta are interactions that bordered the cluster of called positives and that as such still clearly exhibited superior growth than highest background levels.

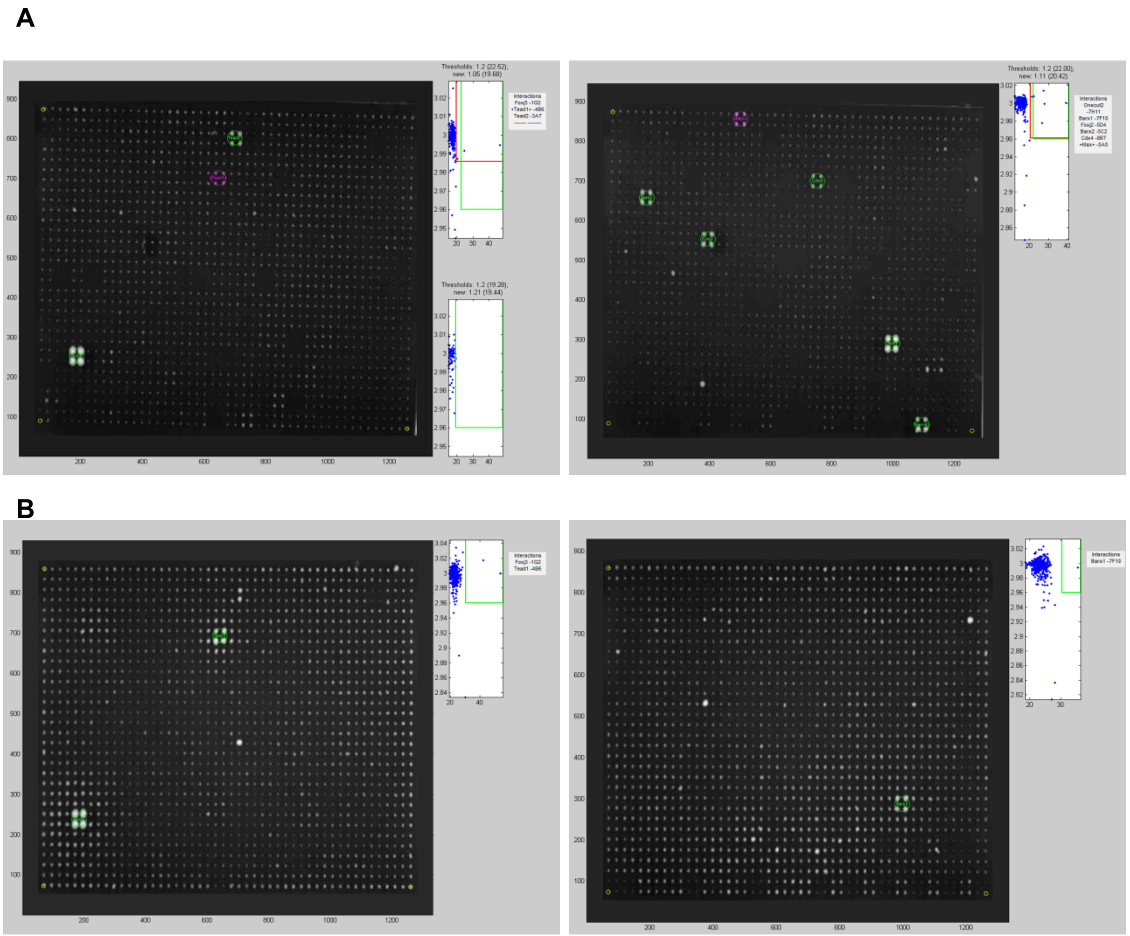

Figure S3 ¦ Y1H-based interactions observed with the *Mlcrhs4* DNA bait based on growth on a selective plate using either (**A**) the direct transformation approach (haploid yeast strains) with 10mM 3-Amino-1,2,4-triazole (3-AT) or (**B**) the mating approach (diploid yeast) with 5mM 3-AT. The identity of each positive interaction can be found in **Supplementary Table S3**. One representative experiment is shown for each Y1H procedure. The green circles represent the interactions scoring 20% above the highest background intensity value (default). Those indicated in magenta are interactions that bordered the cluster of called positives and that as such still clearly exhibited superior growth than highest background levels.

A

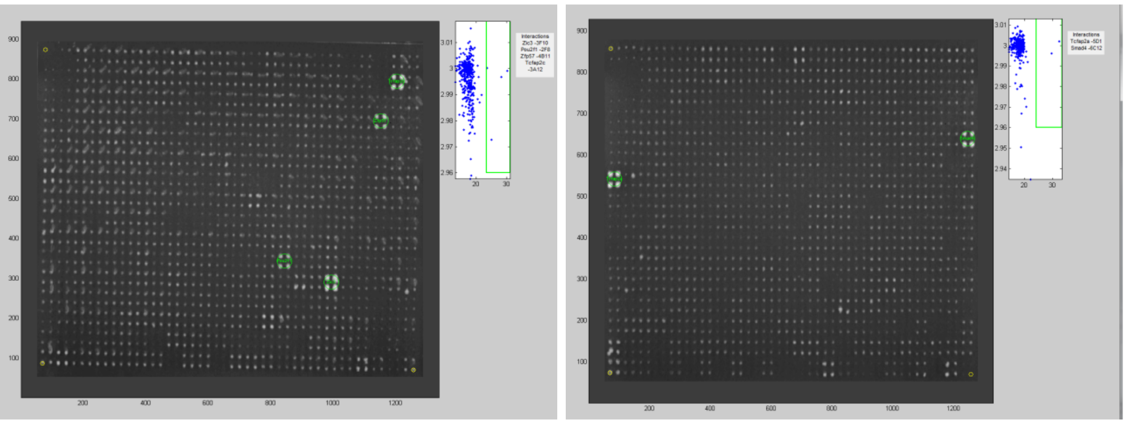

B

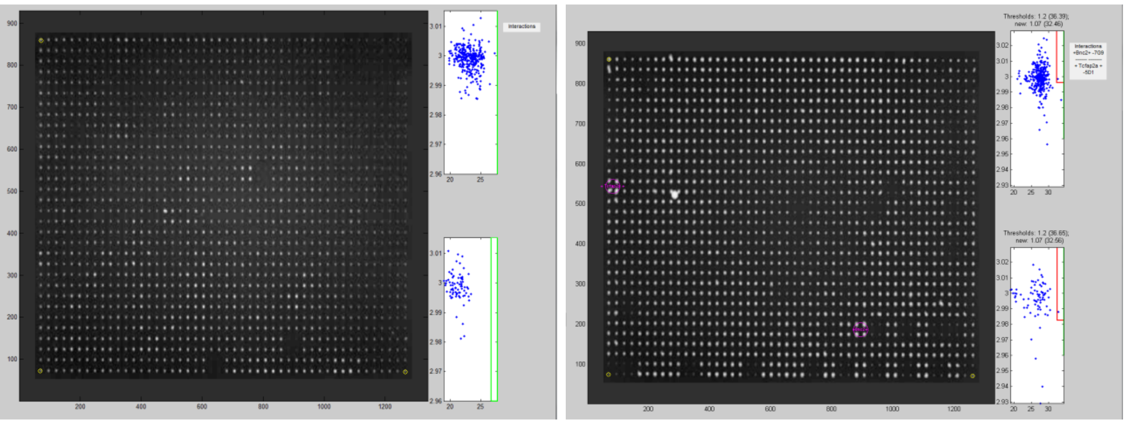

Figure S4 ¦ Y1H-based interactions observed with the *Ptgs2* DNA bait based on growth on a selective plate using either (**A**) the direct transformation approach (haploid yeast strains) with 20mM 3-Amino-1,2,4-triazole (3-AT) or (**B**) the mating approach (diploid yeast) with 5mM 3-AT. Interactions observed with the promoter bait. The identity of each positive interaction can be found in **Supplementary Table S3**. One representative experiment is shown for each Y1H procedure. The green circles represent the interactions scoring 20% above the highest background intensity value (default). Those indicated in magenta are interactions that bordered the cluster of called positives and that as such still clearly exhibited superior growth than highest background levels.

A

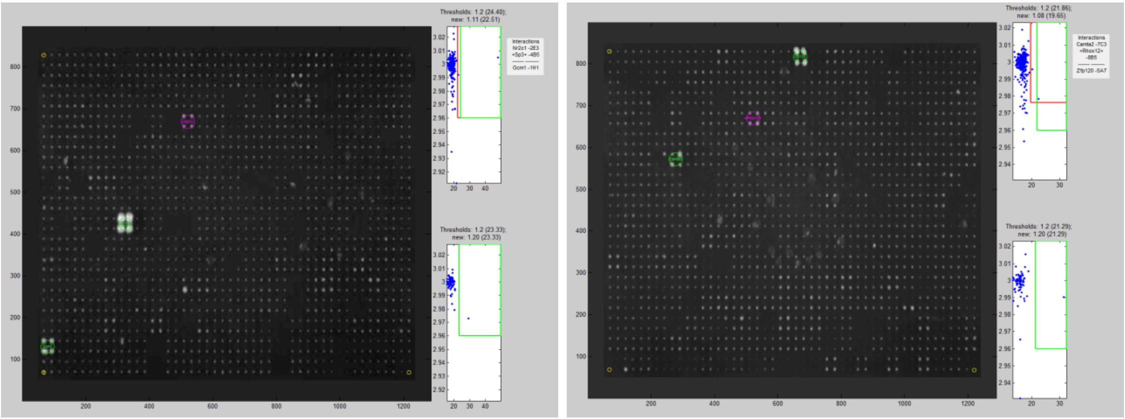

B

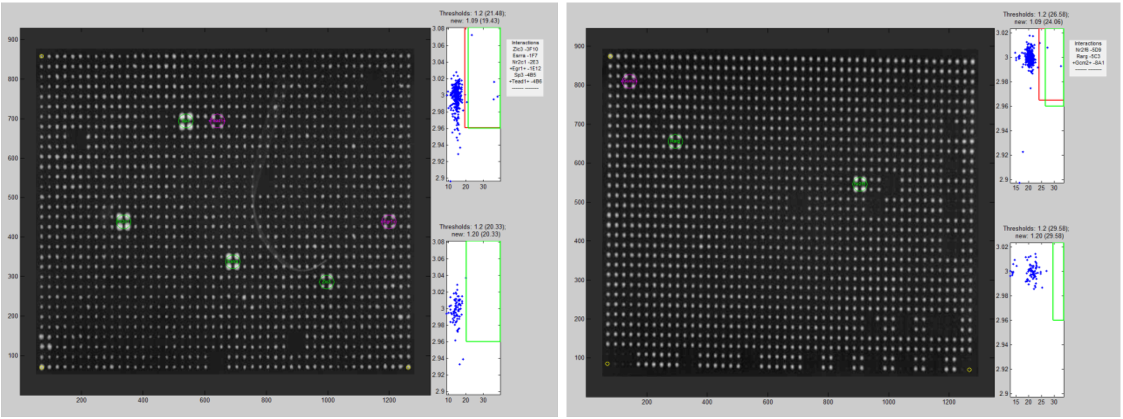

Figure S5 ¦ Y1H-based interactions observed with the *Pou5f1* promoter based on growth on a selective plate using either (**A**) the direct transformation approach (haploid yeast strains) with 40mM 3-Amino-1,2,4-triazole (3-AT) or (**B**) the mating approach (diploid yeast) with 5mM 3-AT. The identity of each positive interaction can be found in **Supplementary Table S3**. One representative experiment is shown for each Y1H procedure. The green circles represent the interactions scoring 20% above the highest background intensity value (default). Those indicated in magenta are interactions that bordered the cluster of called positives and that as such still clearly exhibited superior growth than highest background levels.

A

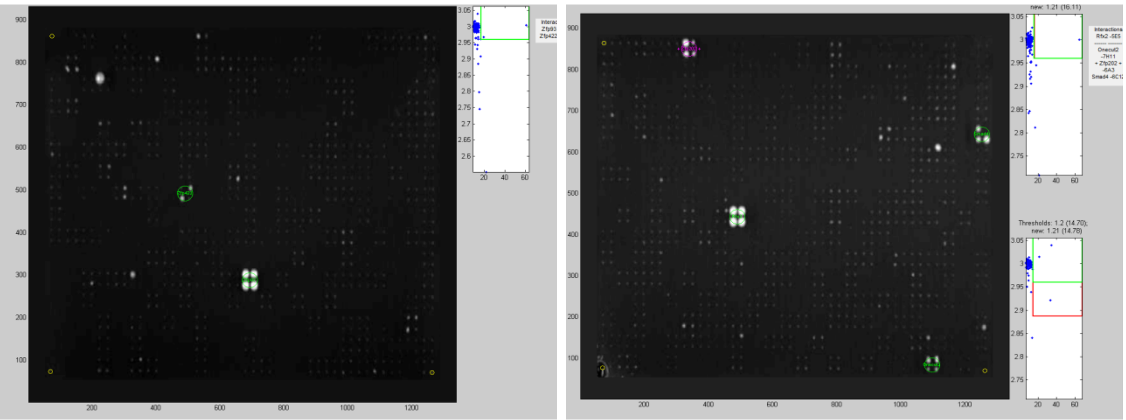

B

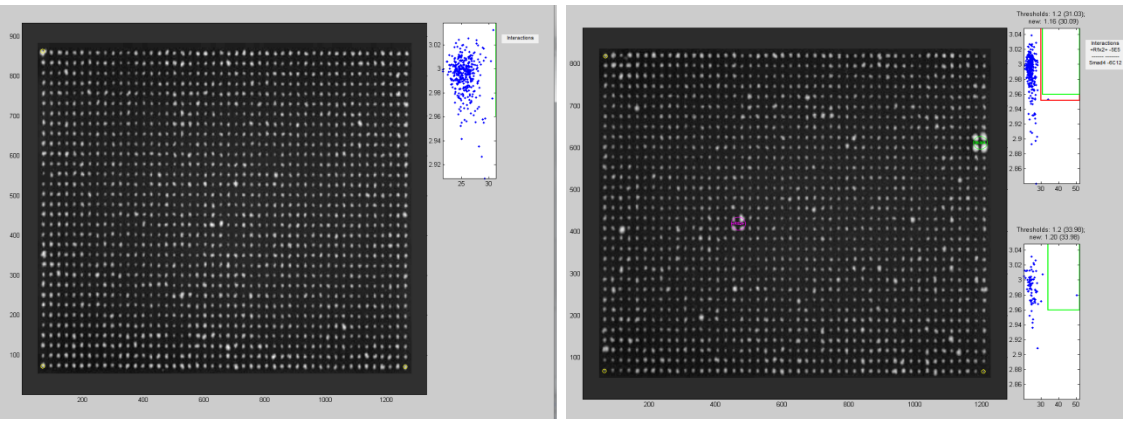

Figure S6 ¦ Y1H-based interactions observed with the *Mcts2-Id1* enhancer bait based on growth on a selective plate using either (**A**) the direct transformation approach (haploid yeast strains) with 20mM 3-Amino-1,2,4-triazole (3-AT) or (**B**) the mating approach (diploid yeast) with 5mM 3-AT. The identity of each positive interaction can be found in **Supplementary Table S3**. One representative experiment is shown for each Y1H procedure. The green circles represent the interactions scoring 20% above the highest background intensity value (default). Those indicated in magenta are interactions that bordered the cluster of called positives and that as such still clearly exhibited superior growth than highest background levels.

A

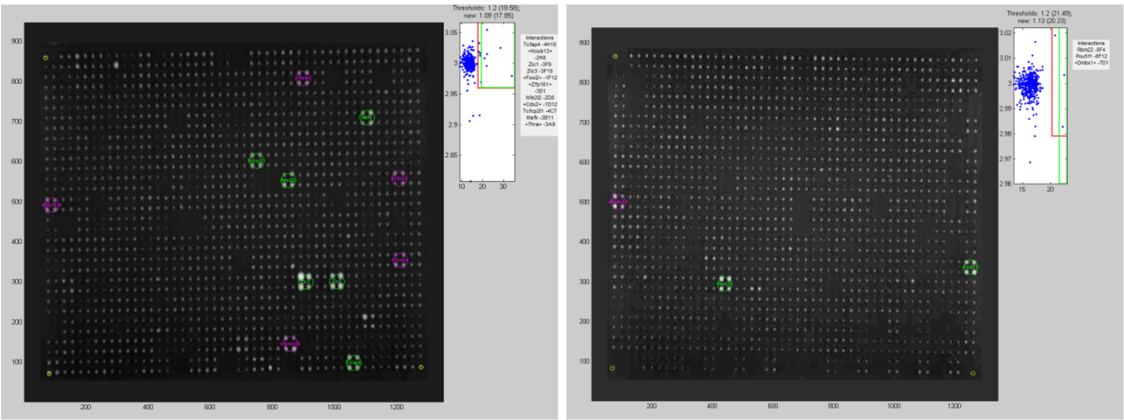

B

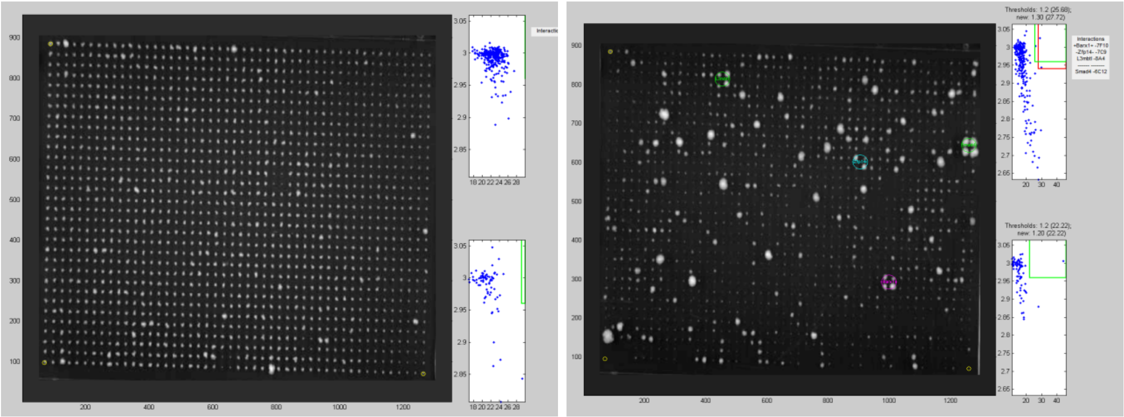

Figure S7 ¦ Y1H-based interactions observed with the *Fos* promoter based on growth on a selective plate using either (**A**) the direct transformation approach (haploid yeast strains) with 10mM 3-Amino-1,2,4-triazole (3-AT) or (**B**) the mating approach (diploid yeast) with 5mM 3-AT. The identity of each positive interaction can be found in **Supplementary Table S3**. One representative experiment is shown for each Y1H procedure. The green circles represent the interactions scoring 20% above the highest background intensity value (default). Those indicated in magenta are interactions that bordered the cluster of called positives and that as such still clearly exhibited superior growth than highest background levels.

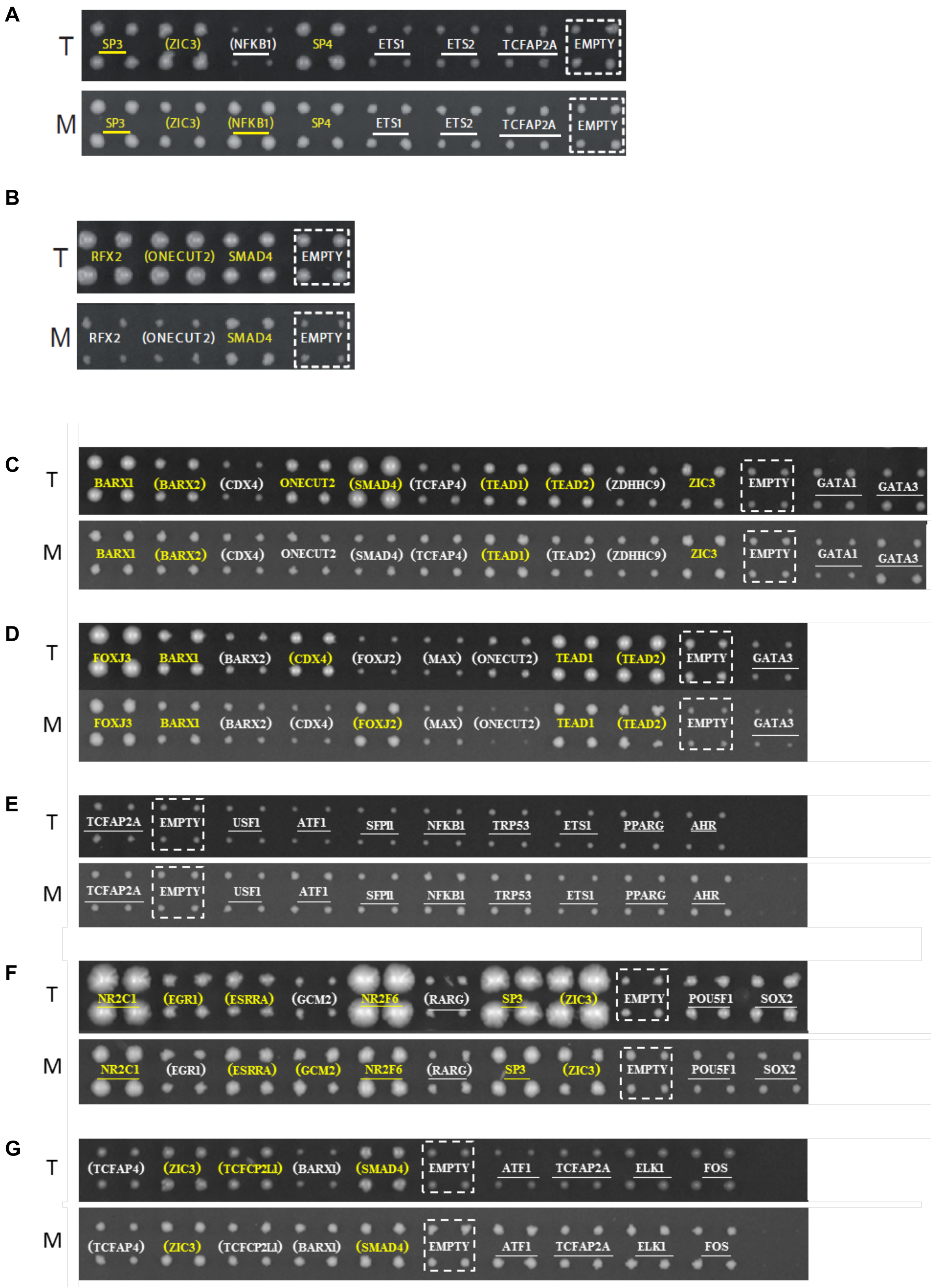

Figure S8 ¦ Manual transformation (T) and mating (M) experiments to validate reproducibly detected interactions detected in the high-throughput Y1H screens with the *Mmp9* promoter (**A**), *Mcts2-Id1* enhancer (**B**), *Rhs7-2* (**C**), *Mlcrhs4* (**D**), *Ptgs2*

promoter (**E**), *Pou5f1* promoter (**F**) and *Fos* promoter (**G**) baits. Yeast strains were spotted in quadruplet. Positive interactions are highlighted in yellow and can be compared to a negative control (original pAD-DEST vector, termed “empty” and indicated via a dashed square) illustrating the background growth for each DNA bait. The known interactors are underlined. The mating or transformation-specific interactors are put in parentheses. The baits are selected on 5mM 3-AT (**A** M; **C** T+M; **D** T+M; **E** T+M; **G** M), 10mM 3-AT (**B** M; **F** M) or 20mM 3-AT (**A** T; **B** T; **F** T; **G** T).

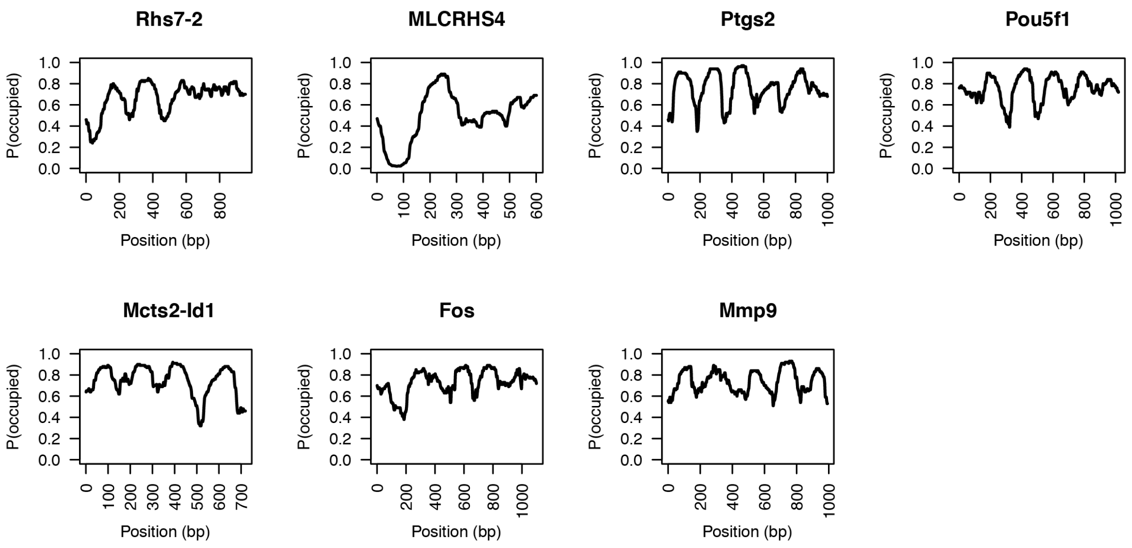

Figure S9 ¦ *In silico* predicted nucleosome occupancy landscape for DNA elements screened with Y1H. See **Methods** for details.

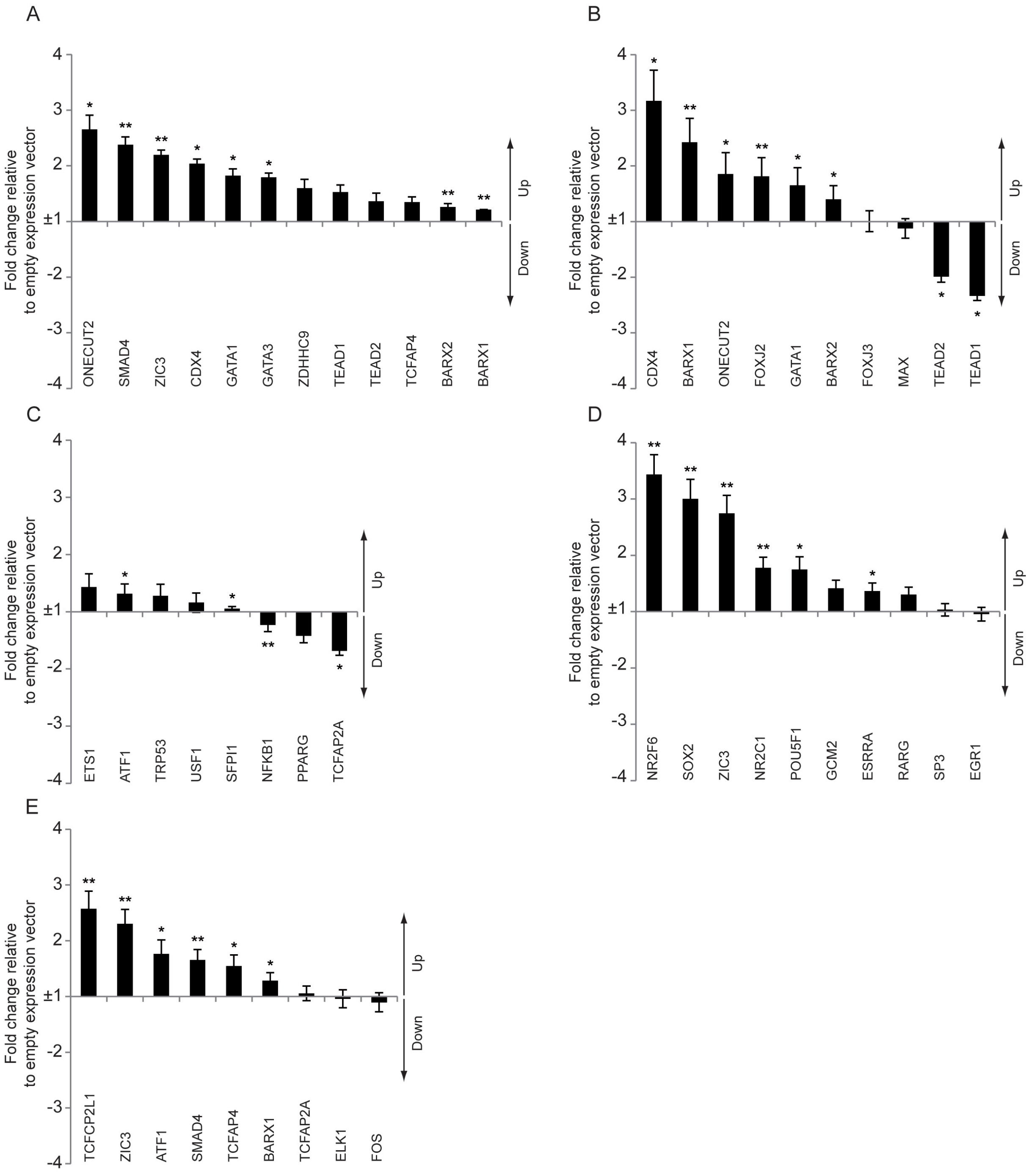

Figure S10 ¦ Luciferase reporter-based validation of consensus interactions with the *Rhs7-2* element (**A**), *Mlcrhs4* element (**B**), *Ptgs2* promoter (**C**), *Pou5f1* promoter (**D**), and the *Fos* promoter (**E**). HEK293 cells were transiently co-transfected with DNA-bait reporter construct, each of the respective TFs and the *Renilla* luciferase vector. The fold change of the normalized firefly to renilla ratio compared to the normalized

firefly to renilla ratio of the negative control (versus the empty expression vector) is plotted. The known interactors reported in the literature (**Supplementary Table S2**) but not found in the Y1H screens, were also tested. The error bars represent the standard error of six independent experiments. $**P < 0.01$ and $0.01 < *P < 0.05$ compared to the negative control.

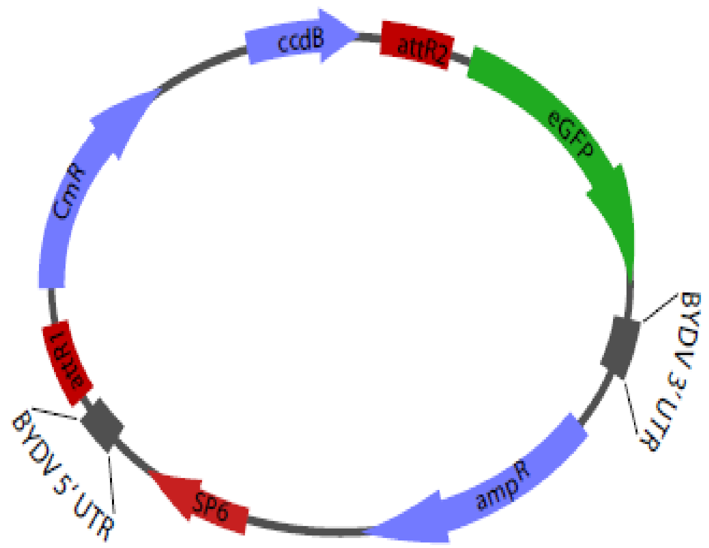

Figure S11 ¦ Schematic representation of the pMARE vector. This vector was generated from the pF3A WG (BYDV) Flexi vector (Promega) by inserting the eGFP coding sequence (EUROSCARF) and the gateway reading frame A cassette (Invitrogen). See **Methods** for more details.

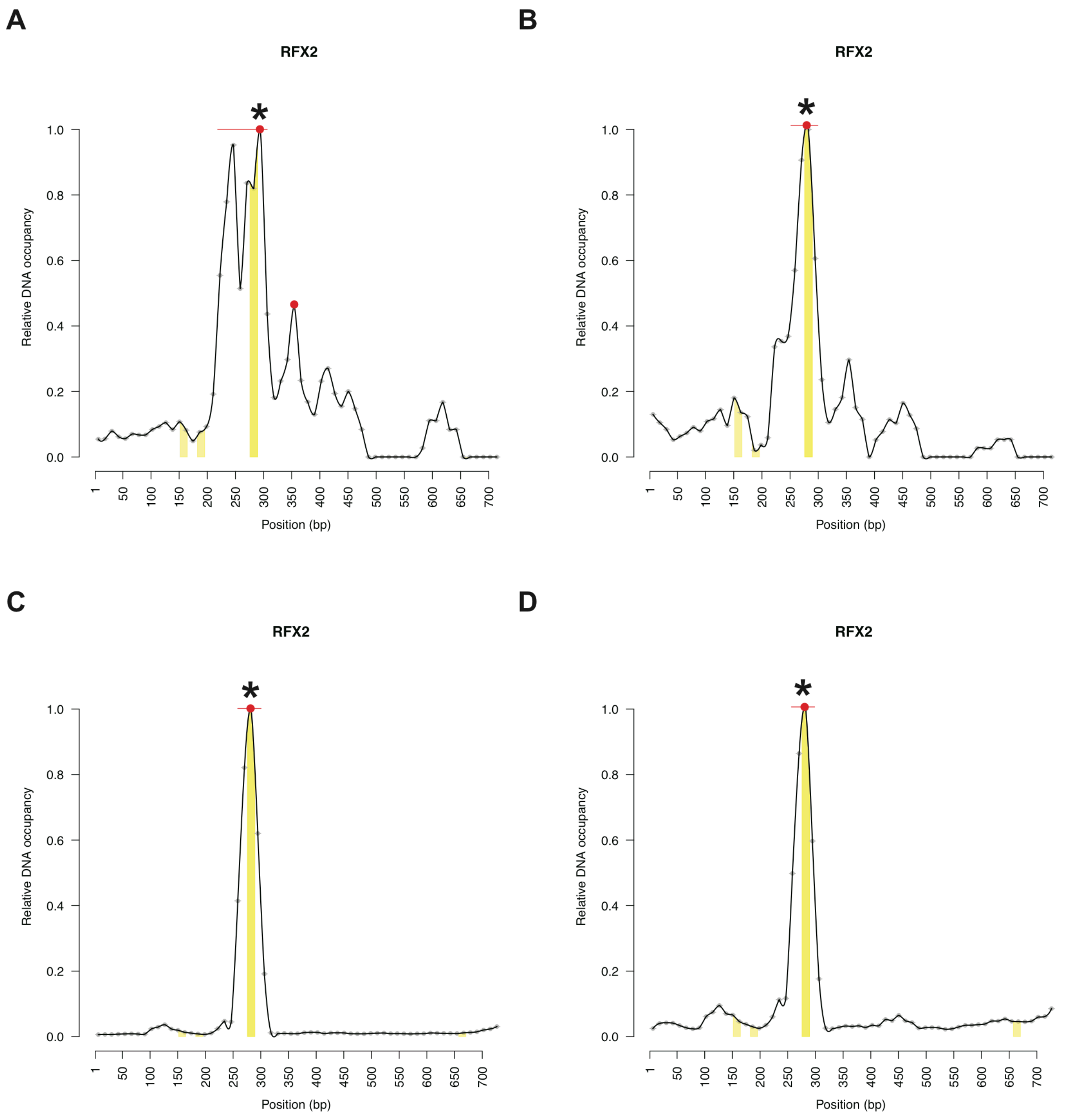

Figure S12 ¦ RFX2 relative DNA occupancy landscapes for the *Mcts2-Id1* enhancer by using two MARE approaches: on-chip protein expression (**A,B**) and off-chip synthesis using a multiplexer design (**C,D**). Bound DNA levels normalized over surface-immobilized protein amounts are plotted for each 12 bp nucleotide stretch as grey dots with horizontal lines indicating the 12 bp region. Signals between every 12 bp nucleotide were estimated by interpolation. Significant peaks are indicated with a red line, peak maxima are indicated with a red dot. Peaks found in both replicates are marked by an asterisk. PWM-based binding site predictions are plotted with yellow bars (FIMO P<1e-3).

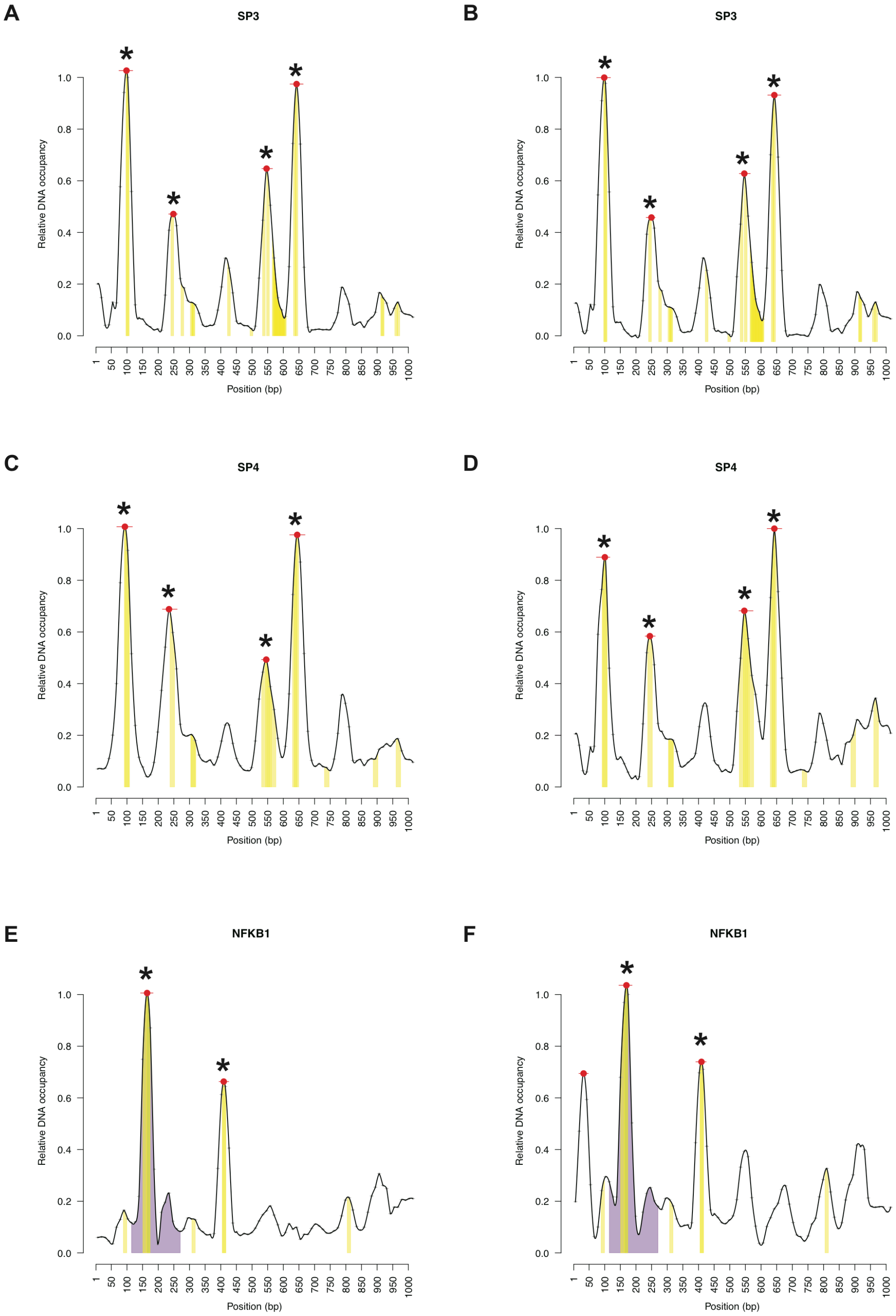
A
SP3
B
SP3
C
SP4
D
SP4
E
NFKB1
F
NFKB1
Relative DNA occupancy
Position (bp)

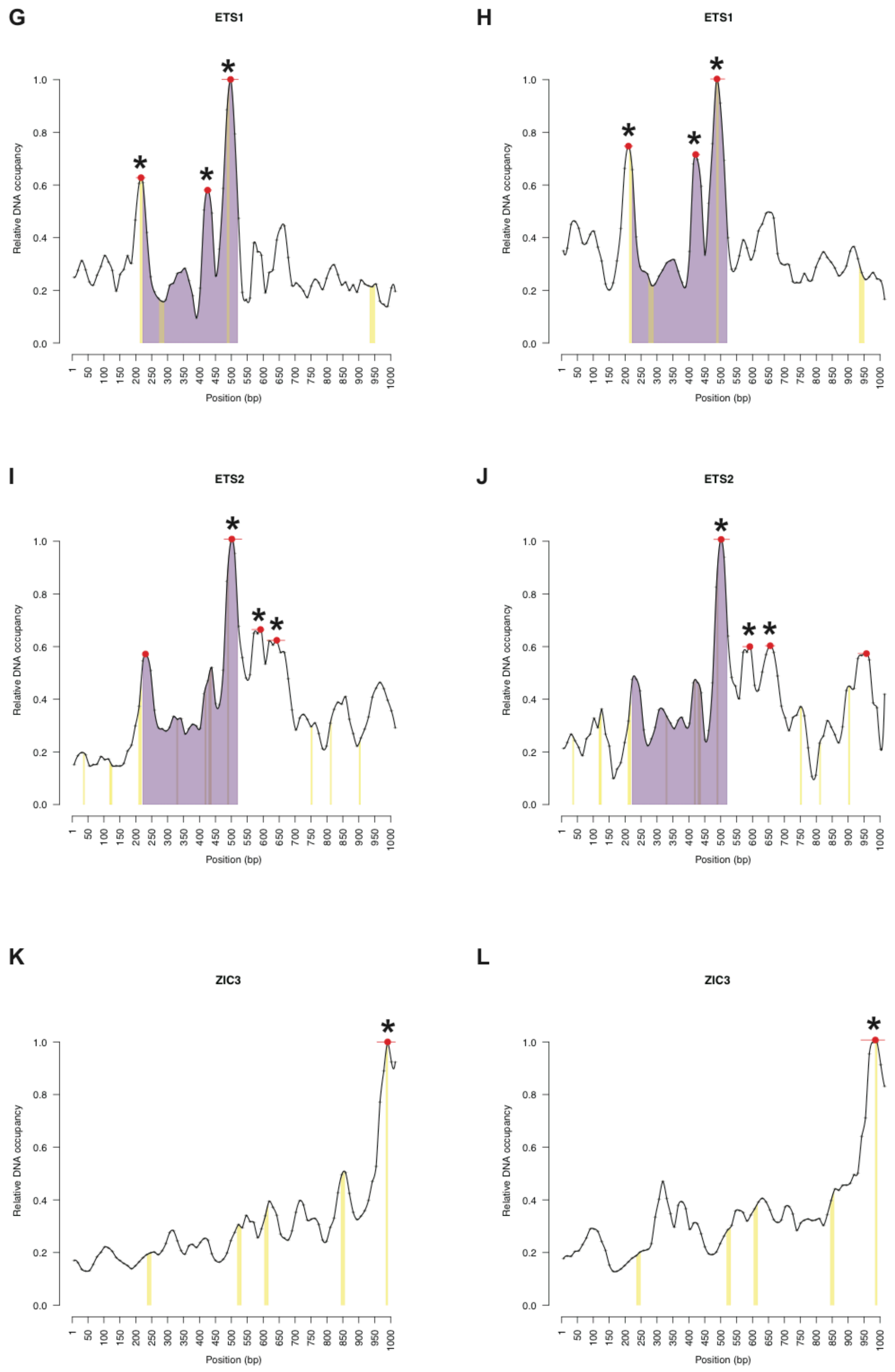

Figure S13 ¦ Identification of specific protein-DNA interactions between the *Mmp9* promoter and Y1H-consensus or positive control TFs. For clarity, the two replicates that are presented in the main manuscript are recapitulated here. Bound DNA levels

normalized over surface-immobilized protein amounts are plotted for each 12 bp nucleotide stretch as grey dots with horizontal lines indicating the 12 bp region. Signals between every 12 bp nucleotide were estimated by interpolation. Significant peaks are indicated with a red line, peak maxima are indicated with a red dot. Peaks found in both replicates are marked by an asterisk. Where available, ChIP-based region, PWM-based binding site predictions (FIMO *P*<1e-3), and consensus binding sites are plotted with purple and yellow bars respectively. TCFAP2A was not analyzed as it failed to express at testable levels. (**A,B**) SP3, (**C,D**) SP4, (**E,F**) NFKB1, (**G,H**) ETS1, (**I,J**) ETS2, (**K,L**) ZIC3.

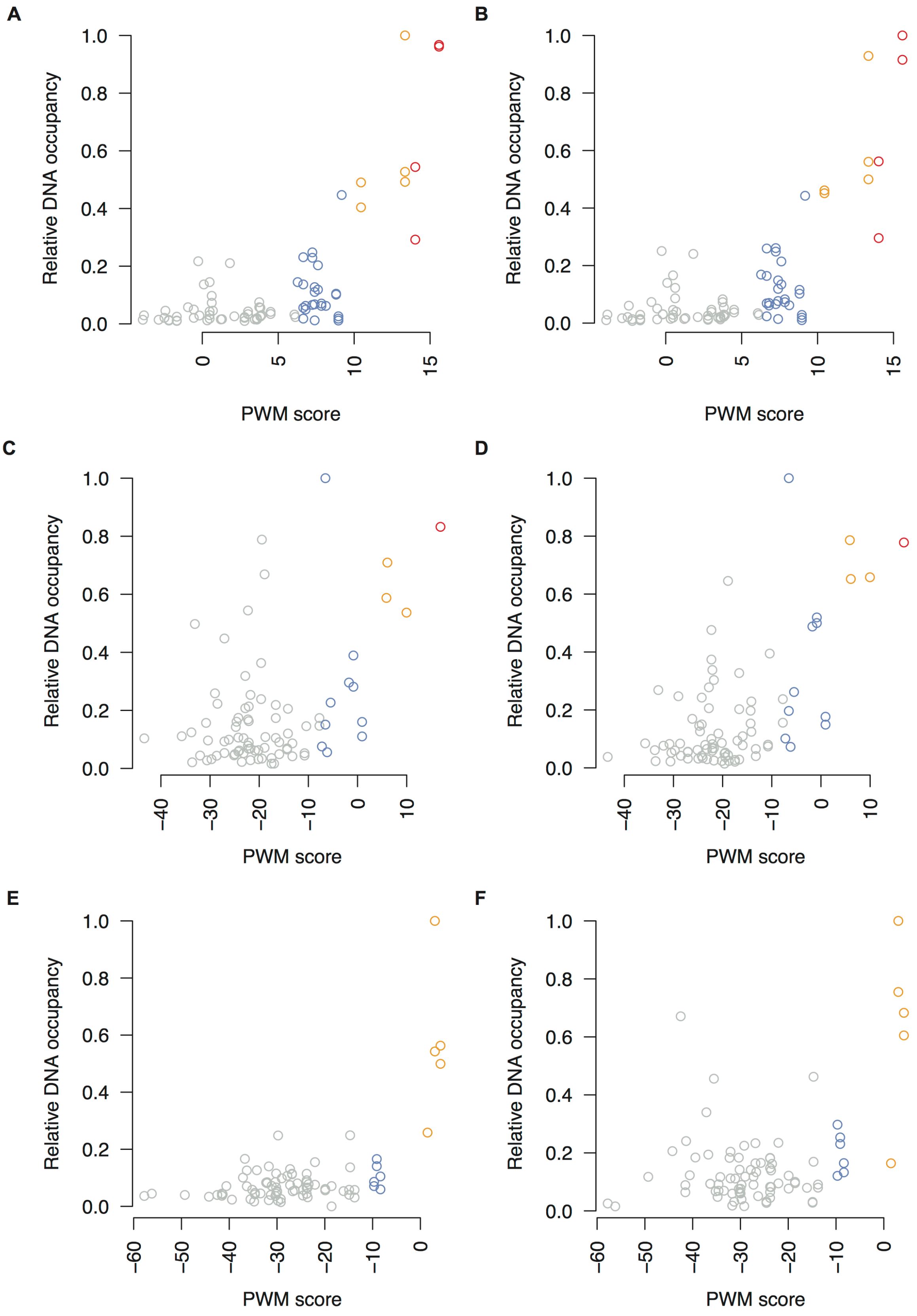
A
B
C
D
E
F
Relative DNA occupancy
PWM score
0.0
0.2
0.4
0.6
0.8
1.0
0
5
10
15
−40
−30
−20
−10
−60
−50

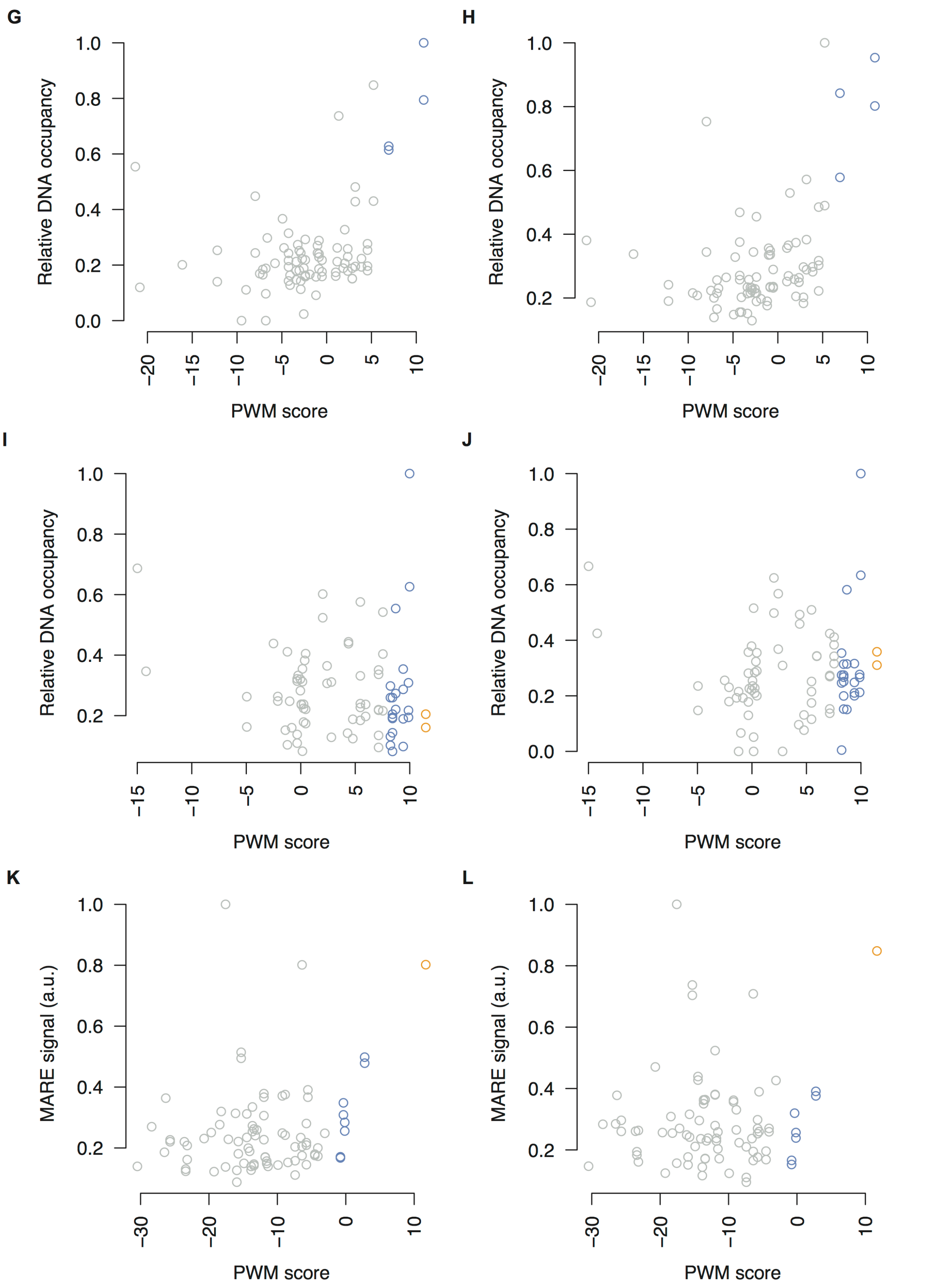

Figure S14 ¦. PWM scores and the relative DNA occupancy landscape of the *Mmp9* promoter. Each data point represents a 36 bp fragment tested with MARE. The PWM

score represents the maximum log-likelihood ratio score per fragment. Two replicates per interaction are shown. Colors indicate motif occurrence probabilities: P<1e-5, red; P<1e-4, orange; P<1e-3, blue; non significant, grey. (**A**,**B**) SP3, (**C**,**D**) SP4, (**E**,**F**) NFKB1, (**G**,**H)** ETS1, (**I**,**J**) ETS2, (**K**,**L**) ZIC3.

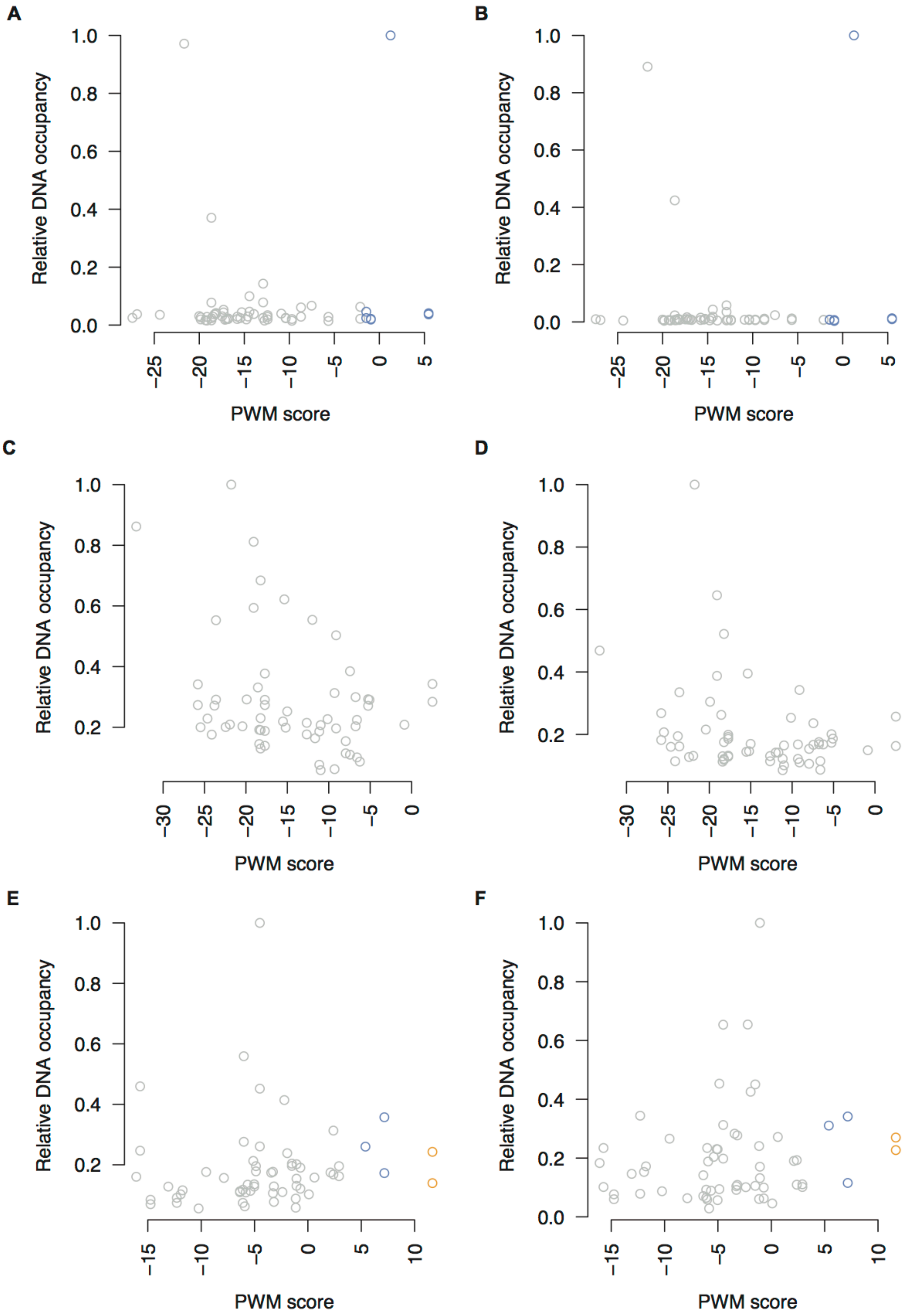

Figure S15 ¦. PWM scores and the relative DNA occupancy landscape of the *Mcts2-Id1* enhancer. Each data point represents a 36 bp fragment tested with MARE. The

PWM score represents the maximum log-likelihood ratio score per fragment. Two replicates per interaction are shown. Colors indicate motif occurrence probabilities: *P*<1e-4, orange; *P*<1e-3, blue; non significant, grey. (**A**,**B**) RFX2, (**C**,**D**) ONECUT2, (**E**,**F**) SMAD4.

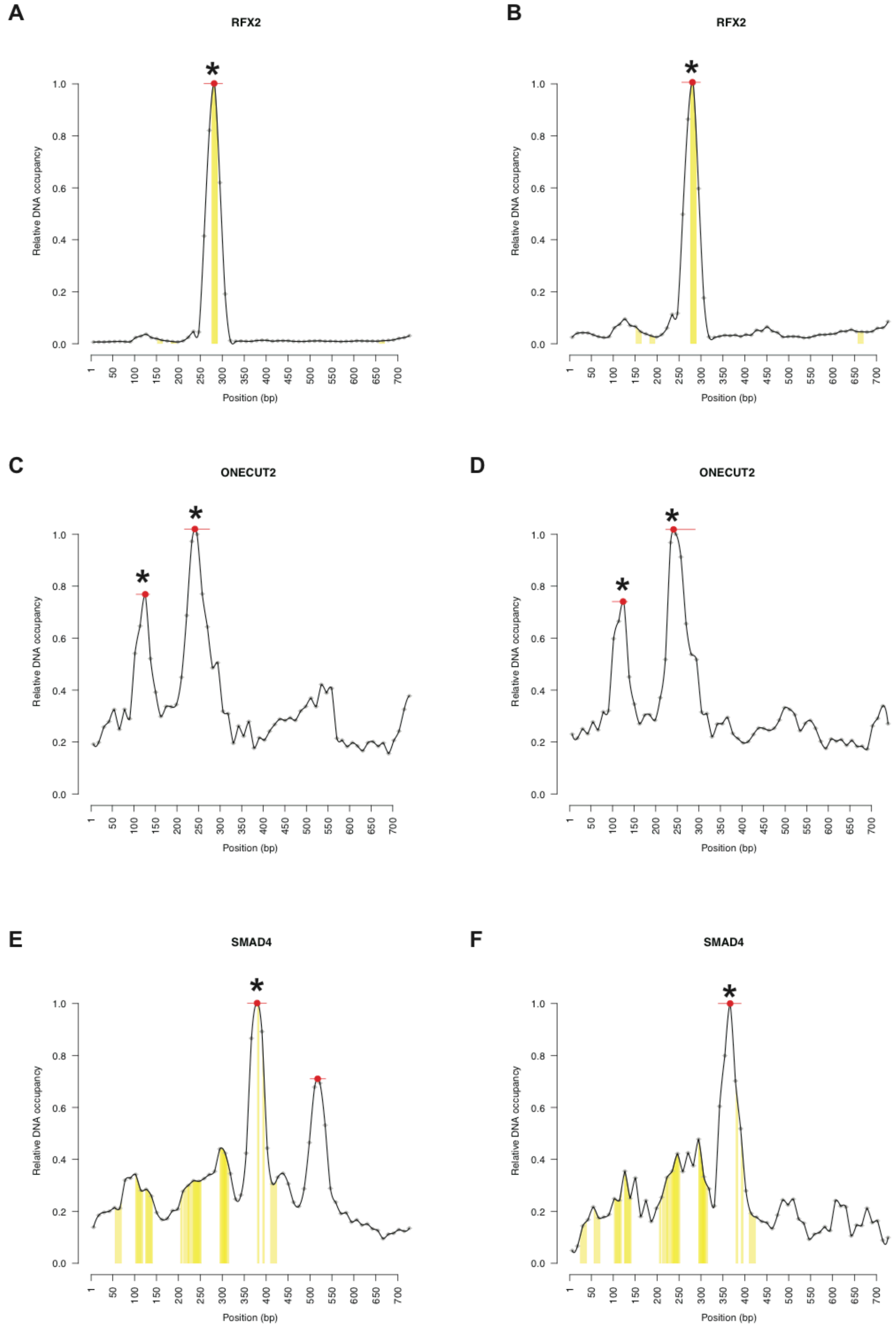

Figure S16 ¦ Identification of specific protein-DNA interactions between the *Mcts2-Id1* enhancer and Y1H-consensus TFs. For clarity, the replicates that are presented in the main manuscript and those involving RFX2 as shown in **Figure S12** are

recapitulated here. Bound DNA levels normalized over surface-immobilized protein amounts are plotted for each 12 bp nucleotide stretch as grey dots with horizontal lines indicating the 12 bp region. Signals between every 12 bp nucleotide were estimated by interpolation. Significant peaks are indicated with a red line, peak maxima are indicated with a red dot. Peaks found in both replicates are marked by an asterisk. PWM-based binding site predictions are indicated with yellow bars (FIMO P<1e-3). (**A,B**) RFX2, (**C,D**) ONECUT2, (**E,F**) SMAD4.

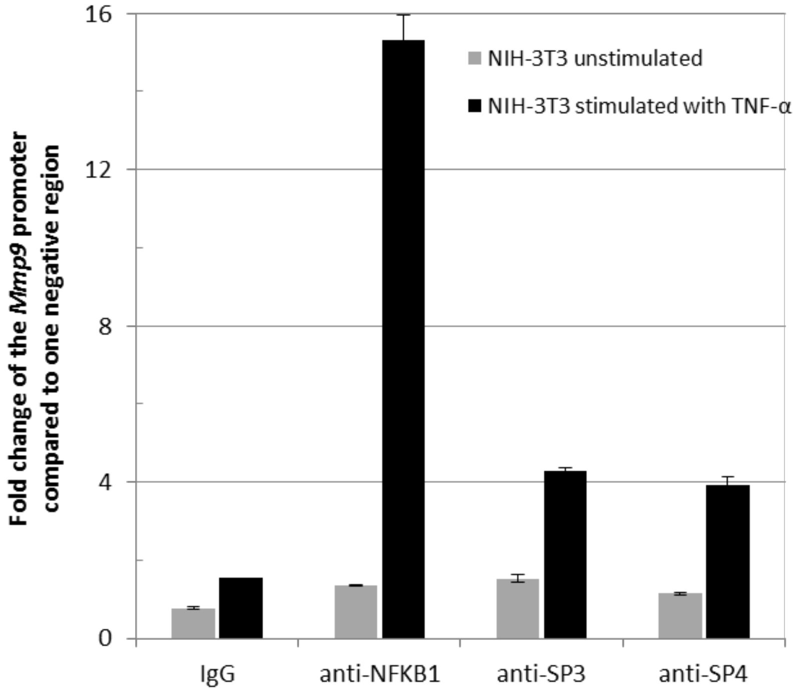

Figure S17 ¦ ChIP-qPCR analysis of SP3, SP4, and NFKB1 binding to the *Mmp9* promoter in TNF-α stimulated versus unstimulated NIH-3T3 cells. NIH-3T3 cells were crosslinked, lysed, and proteins immunoprecipitated using anti-NFKB1, anti-SP3, and anti-SP4 antibodies (**Methods**). Rabbit IgG was used as a negative control. The co-precipitated DNA was analyzed by qPCR with primers targeting the *Mmp9* promoter and one negative control region. The fold-change between these two regions is plotted. The error bars represent the standard error of two independent experiments.

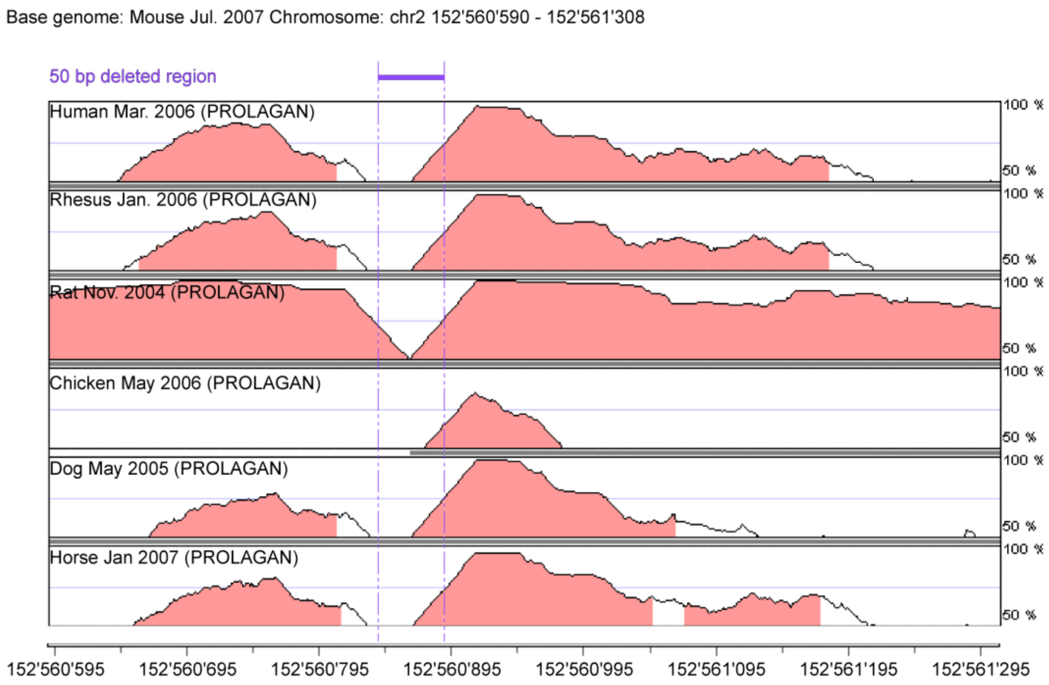

Figure S18 ¦ Comparative sequence analysis of the *Mcts2-Id1* enhancer using the VISTA browser. Regions of high conservation are colored in pink (non-coding regions). The deleted region of 50bp is highlighted using purple lines, revealing that this segment shows lower conservation than its surrounding segments.

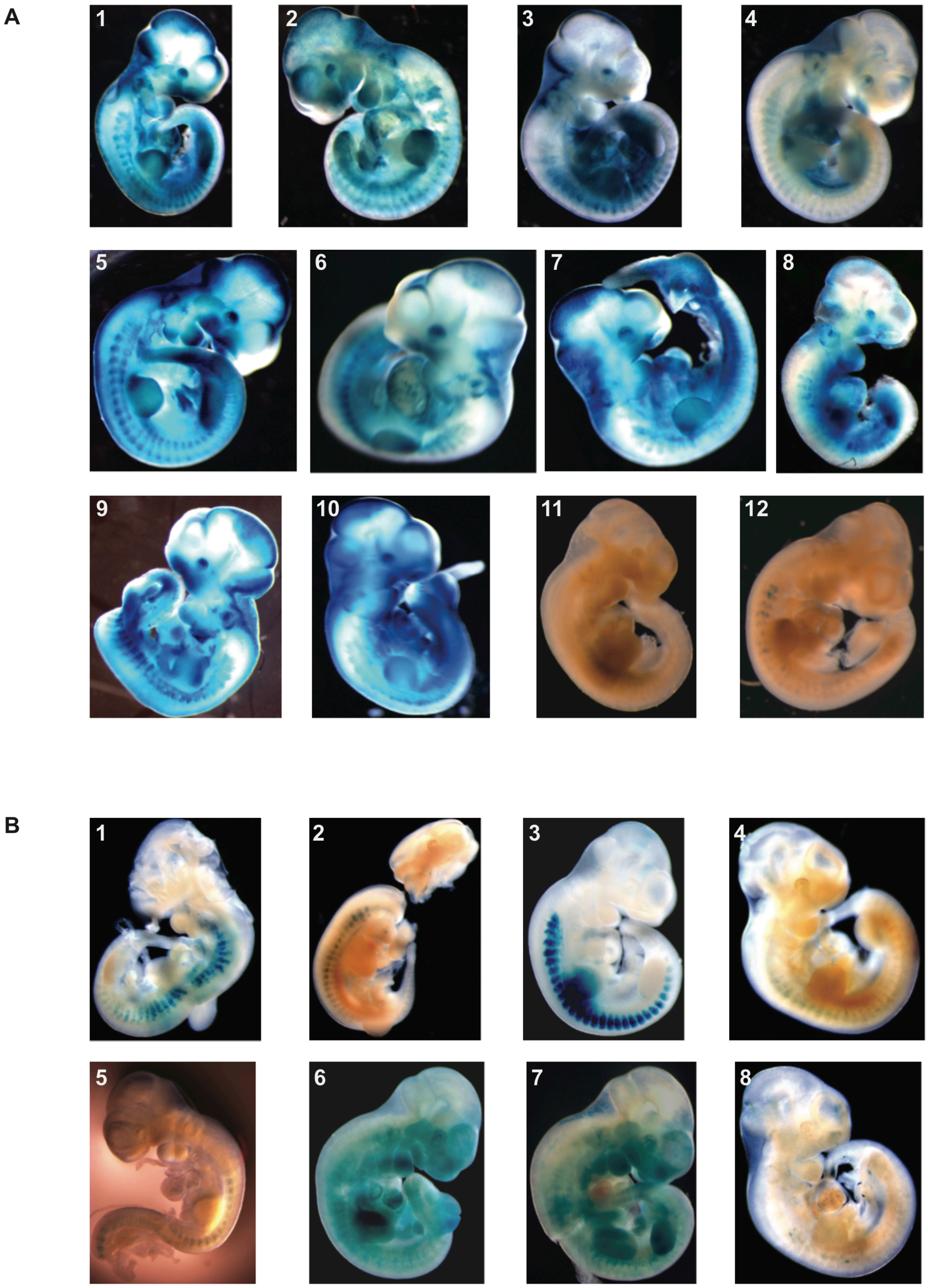
A
1
2
3
4
5
6
7
8
9
10
11
12
B
1
2
3
4
5
6
7
8

C.

**Constructs**	**Total number of embryos**	**Number of transgenic embryos**	**Number of embryos sharing the same *LacZ* pattern**
***Mcts1-Id1* enhancer**	23	19	9/11
***Mcts1-Id1* enhancer Δ50**	29	11	5/8

Figure S19 ¦ *LacZ* staining results of transgenic embryos bearing the *Mcts1-Id1* enhancer construct (**A**) or the *Mcts1-Id1* enhancer deletion construct (50bp, **B**) at E 10.5. The presented embryos **A1-A9** and **B1-B5** are the ones showing specific and reproducible expression patterns as explained in the main text. Pictures of non-transgenic embryos and those that stained completely blue due to active region trapping are not shown. (**C**) Table summarizing the results of the in vivo enhancer activity analysis experiments.

Supplementary Tables

Supplementary Table S1. Predicted TFs in the *Mus musculus* genome and their cloning status.

Supplementary Table S2. List of DNA baits used in this study as well as their reported TF interactors.

Supplementary Table S3. Summary of all the interactors found in the Y1H transformation, Y1H mating, and luciferase assays.

Supplementary Table S4. Evaluation of Y1H-, luciferase-, and literature-based TF-DNA interactions with PWM-based binding site predictions.

Supplementary Table S5. Nucleosome positioning and Y1H performance.

Supplementary Table S6. Motif load and Y1H performance.

Supplementary Table S7. Predicted MARE binding sites without motif scanning support.
